# Doença Cardíaca Isquêmica e Nível de Renda – Uma Reflexão Acerca de Determinantes Sociais e Estruturais

**DOI:** 10.36660/abc.20240014

**Published:** 2024-04-09

**Authors:** Otávio Azevedo Bertoletti

**Affiliations:** 1 Hospital de Clínicas de Porto Alegre Porto Alegre RS Brasil Hospital de Clínicas de Porto Alegre, Porto Alegre, RS – Brasil

**Keywords:** Doença Cardíaca Isquêmica, Nível de Renda, Caminhabilidade, Determinantes Sociais da Saúde

Determinantes sociais e estruturais da saúde têm ganhado destaque quanto a sua influência na saúde cardiovascular da população. Em recentes publicações, a *American Heart Association* tem dirigido sua atenção às desigualdades nos cuidados de saúde, mediadas por determinantes sociais (como renda, educação, segurança, local de moradia e vizinhança) e estruturais (acesso à saúde, saneamento básico, ar limpo, ambiente saudável), e seus impactos deletérios na saúde cardiovascular.^1-3^ Discute como implementar ações com potencial para eliminar essas desigualdades no cuidado de saúde que afetam desfavoravelmente a saúde cardiovascular^3^ das populações marginalizadas.^2^ Além disso, aborda estratégias para atingir uma saúde cardiovascular para todos, de forma equânime, com foco no aumento médio da longevidade populacional,^4^ haja vista que as doenças cardiovasculares continuam sendo a principal causa de morte mundialmente, inclusive no Brasil, liderada pela doença cardíaca isquêmica.^5^

Nessa direção, o estudo transversal de Cerci et al.,^6^ vem a contribuir na análise se um ambiente público propício a caminhadas (walkability) na vizinhança medeia a potencial relação entre nível de renda e doença isquêmica do coração (DIC) de residentes de uma cidade de 1.773.718 habitantes,^7^ inserida num país de renda mediana. Os autores analisaram 26.415 indivíduos da cidade de Curitiba/Brasil, no período entre 2010 e 2017, sendo 96,5% cobertos por plano privado de saúde, diagnosticados com DIC através de exame de imagem de perfusão miocárdica por tomografia computadorizada com emissão única de fótons (SPECT-MPI).

Por meio da geolocalização e cruzamento com dados censitários da região de domicílio identificaram e cruzaram dados de renda, escolaridade e nível de criminalidade. E, assim, analisaram as características socioeconômicas dos participantes. Os autores encontraram uma associação inversa e independente entre nível de renda e prevalência de DIC, odds ratio = 0,91 (IC de 95%: 0,87 a 0,96).

Embora a caminhabilidade, mensurada através da combinação de 4 variáveis – conectividade viária, densidade residencial, densidade comercial e uso misto do solo –, tenha apresentado associação direta com os níveis de renda – os setores censitários de maior renda apresentaram associação com uma maior caminhabilidade 1,79 (IC 95%: 1,49 a 2,08) –, ela não mediou a associação entre nível de renda e DIC (percentual de mediação= -0,3%).

Secundariamente, este estudo corroborou os dados da literatura,^8^ demonstrando significância estatística na relação entre nível de renda e prevalência de fatores de risco. Identificaram que quanto menor a renda, maior a prevalência de pessoas fisicamente inativas, tabagistas, hipertensas e com diabetes.

Em que pese o desenho deste estudo não tenha se fundamentado numa análise probabilística de representação populacional e, portanto, tem limitações na extrapolação de seus resultados para esse fim, os autores evidenciam análises relevantes acerca da saúde de residentes de um grande centro urbano. Concluem que residir em um setor censitário de baixa renda foi independentemente associado a maior prevalência de DIC, independente do sexo. Vizinhanças com estrutura que não favorece a caminhabilidade são ocupadas por pessoas de menor renda, apesar de não ter sido evidenciado que a caminhabilidade possa influenciar a relação entre nível de renda e DIC.
