# Supplementary material for: Doença Cardíaca Isquêmica e Nível de Renda – Uma Reflexão Acerca de Determinantes Sociais e Estruturais
Source: Arq Bras Cardiol. 2024 Apr 9;120(11):e20240014. [Article in Portuguese] doi: 10.36660/abc.20240014 (PMC11098580; doi:10.36660/abc.20240014)
Supplement: Translation

# Translation: Ischemic Heart Disease and Income Level – A Thinking on Social and Structural Determinants

**Authors:** Otávio Azevedo Bertoletti

**Affiliations:** 1 Porto Alegre RS Brasil Hospital de Clínicas de Porto Alegre, Porto Alegre, RS – Brasil

**Keywords:** Ischemic Heart Disease, Income Level, Walkability, Health Social Determinants

Social and structural determinants of health have gained prominence regarding their influence on the cardiovascular health of the population. In recent publications, the American Heart Association has directed its attention to inequalities in healthcare, mediated by social (such as income, education, safety, place of residence, and neighborhood) and structural determinants (access to healthcare, basic sanitation, clean air, healthy environment), and its deleterious impacts on cardiovascular health.^1-3^ It discusses how to implement actions with the potential to eliminate these inequalities in health care that adversely affect the cardiovascular health^3^ of marginalized populations.^2^ In addition, it addresses strategies to achieve cardiovascular health for all in an equitable way, with a focus on increasing the average population longevity,^4^ given that cardiovascular diseases continue to be the main cause of death worldwide, including in Brazil, led by ischemic heart disease.^5^.

In this direction, the cross-sectional study by Cerci et al.^6^ contributes to the analysis of whether a public environment conducive to walking (walkability) in the neighborhood mediates the potential relationship between income level and ischemic heart disease (IHD) of residents of a city with 1,773,718 inhabitants,^7^ located in a middle-income country. The authors analyzed 26,415 individuals from the city of Curitiba/Brazil, in the period between 2010 and 2017, 96.5% of whom were covered by a private health plan and diagnosed with IHD through myocardial perfusion imaging using computed tomography with single photon emission. (SPECT-MPI).

Through geolocation and cross-referencing with census data from the region of residence, they identified and cross-referenced data on income, education, and level of crime. So, they analyzed the socioeconomic characteristics of the participants. The authors found an inverse and independent association between income level and IHD prevalence, odds ratio = 0.91 (95% CI: 0.87 to 0.96).

Although walkability, measured through the combination of 4 variables – road connectivity, residential density, commercial density, and mixed land use – has shown a direct association with income levels – the census tracts with higher income have been associated with greater walkability 1 .79 (95% CI: 1.49 to 2.08) – it did not mediate the association between income level and IHD (percentage of mediation = -0.3%).

Secondarily, this study corroborated data from the literature,^8^ demonstrating statistical significance in the relationship between income level and prevalence of risk factors. They identified that the lower the income, the higher the prevalence of physically inactive people, smokers, hypertensive people, and people with diabetes.

Despite the fact that the design of this study was not based on a probabilistic analysis of population representation and, therefore, has limitations in extrapolating its results for this purpose, the authors highlight relevant analyses regarding the health of residents of a large urban center. They conclude that living in a low-income census tract was independently associated with a higher prevalence of IHD, regardless of sex. Neighborhoods with structures that do not favor walkability are occupied by people with lower incomes, although there has been no evidence that walkability can influence the relationship between income level and IHD.
